# Risk factors for kidney stone disease recurrence: a comprehensive meta-analysis

**DOI:** 10.1186/s12894-022-01017-4

**Published:** 2022-04-19

**Authors:** Kai Wang, Jing Ge, Wenlong Han, Dong Wang, Yinjuan Zhao, Yanhao Shen, Jiexun Chen, Dongming Chen, Jing Wu, Ning Shen, Shuai Zhu, Bin Xue, Xianlin Xu

**Affiliations:** 1 Department of Urology, Sir Run Run Hospital, Nanjing Medical University, 109 Longmian Road, Jiangning District, Nanjing, 211100 Jiangsu Province China; 2 Department of Core Laboratory, Sir Run Run Hospital, Nanjing Medical University, 109 Longmian Road, Jiangning District, Nanjing, 211100 Jiangsu Province China; 3 Collaborative Innovation Center of Sustainable Forestry in Southern China, College of Forestry, Nanjing Forestry University, Nanjing, 210037 Jiangsu Province China; 4 China Exposomics Institute (CEI) Precision Medicine Co. Ltd, Shanghai, 200120 China

**Keywords:** Kidney stone disease, Meta-analysis, Recurrence, Risk factor

## Abstract

**Background:**

Kidney stone disease (KSD) is a common illness that causes an economic burden globally. It is easy for patients to relapse once they have suffered from this disease. The reported recurrence rate of KSD ranged from 6.1% to 66.9%. We performed this meta-analysis to identify various potential risk factors for the recurrence of KSD.

**Methods:**

The PubMed, Embase and Web of Science databases were searched using suitable keywords from inception to Mar 2022. A total of 2,663 records were collected initially. After screening the literature according to the inclusion and exclusion criteria, 53 articles (40 retrospective studies; 13 prospective studies) including 488,130 patients were enrolled. The study protocol was registered with PROSPERO (No. CRD42020171771).

**Results:**

The pooled results indicated that 12 risk factors including younger age (n = 18), higher BMI (n = 16), family history of kidney stones (n = 12), personal history of kidney stones (n = 11), hypertension (n = 5), uric acid stone (n = 4), race of Caucasian (n = 3), suspected kidney stone episode before the first confirmed stone episode (n = 3), surgery (n = 3), any concurrent asymptomatic (nonobstructing) stone (n = 2), pelvic or lower pole kidney stone (n = 2), and 24 h urine test completion (n = 2) were identified to be associated with KSD recurrence. In the subgroup analysis, patients with higher BMI (OR = 1.062), personal history of nephrolithiasis (OR = 1.402), or surgery (OR = 3.178) had a higher risk of radiographic KSD recurrence.

**Conclusions:**

We identified 12 risk factors related to the recurrence of KSD. The results of this analysis could serve to construct recurrence prediction models. It could also supply a basis for preventing the recurrence of KSD.

**Supplementary Information:**

The online version contains supplementary material available at 10.1186/s12894-022-01017-4.

## Background

Kidney stone disease (KSD) is a common issue with a high health care burden that affects the quality of life among the global population. The incidence rate of nephrolithiasis increases annually, estimated to be 14% in England and 10.1% in the United States [1, 2]. Its etiology is multifactorial and includes age, sex, geography, climate, race, dietary, genetic factors and so on [3]. Approximately half of the patients with nephrolithiasis will undergo a second episode of renal colic within 10 years [4]. More than 10% of patients could experience more relapses [5]. The probability of symptomatic stone recurrence in children reached 50% within 3 years [6]. Additionally, the recurrence rate of urinary calculi in patients with specific stone mineral compositions and morphologies can even be up to 82.4% [7].

The recurrence of KSD varies greatly among different patients. Some patients have nephrolithiasis only once, while others have frequent recurrences. Although preventive measures such as diet and drugs have been implemented and have achieved significant results, the effectiveness of these interventions is still limited [8, 9]. Identifying risk factors for relapse of KSD can help clinicians develop better preventive intervention plans for patients.

Existing studies have only summarized limited risk factors for KSD recurrence [10, 11]. Nevertheless, KSD recurrence is likely associated with several different risk factors. When multiple risk factors are present, systematic evaluation is positive for individualized treatment. In addition, the relationships reported in the existing studies between some known risk factors and kidney stone recurrence are inconsistent [12]. Thus, the aim of this meta-analysis was to comprehensively explore various potential risk factors for the recurrence of KSD.

## Methods

### Search strategy

The Preferred Reporting Items for Systematic Reviews and Meta-Analyses (PRISMA) and Meta-analysis of observational studies in epidemiology (MOOSE) guidelines were utilized when this meta-analysis was conducted [13]. The PubMed, Embase and Web of Science databases were searched to identify the studies that determined the association between various risk factors and recurrence of KSD. The keywords used were ‘Nephrolithiasis’ OR ‘Nephrolith’ OR ‘Kidney Calculus’ OR ‘Kidney Stones’ OR ‘Kidney Stone’ OR ‘Renal Calculi’ (all fields) AND ‘Relapse’ OR ‘Relapses’ OR ‘Recurrences’ OR ‘Recrudescence’ OR ‘Recrudescences’ (all fields) AND ‘risk factor’ OR ‘association’ OR ‘relative risk’ OR ‘odds ratio’ OR ‘Populations at Risk’ (all fields). The complete Boolean formula regarding the keywords and search hits is shown in Additional file 1: Table S1. Two investigators (KW and JG) independently performed the retrieval on Mar 11, 2022. The references of the identified papers were also screened to determine further potential studies. This study protocol was registered with PROSPERO (No. CRD42020171771).

### Selection criteria

Eligible studies were screened according to the following criteria: (1) any prospective or retrospective study reported the risk factors for recurrence of KSD; (2) sufficient data to estimate the odds ratio (OR), relative risk (RR), or hazard ratio (HR) and their 95% confidence intervals (CIs) reported according to the risk factors; and (3) only complete or the latest studies were included in several studies reported the same risk factors in the same cohort. The recurrence of KSD was defined as the symptomatic, radiographic appearance, or repeated interventions of stones. Reviews, case reports, nonhuman trials, letters, conference abstracts and comments were excluded. Cross-sectional studies were excluded. Studies whose control groups contained healthy subjects or sample sizes were < 40 or lacked key data were also excluded. If only the Kaplan–Meier curves of risk factors for recurrence of KSD were available, we extracted the HR and 95% CI data. The titles and abstracts of all literature were first independently screened by two authors. Further evaluation was conducted by browsing the full texts. Any disagreement was eventually resolved.

### Data extraction and quality assessment

DMC and YHS independently extracted the data required from all eligible studies. JW and DW assessed the quality of each study according to the Newcastle–Ottawa Quality Assessment Scale (NOS) as described in our previous work [14, 15]. Information on the first author’s surname, publication year, population characteristics, sample size, follow-up time, the recurrence rate of KSD, and risk factors for recurrence of KSD.

### Statistical analysis

Any RR and HR with similar values were merged into OR. Pooled ORs and their 95% CIs were used to describe the relationship between various risk factors and recurrence of KSD. A minimum of 2 studies for a risk factor were analyzed. Heterogeneity was assessed by Cochran’s Q test and Higgins’ I-squared statistics. When *I*^2^ > 50% and/or *P* < 0.1, a random-effects model was used. Otherwise, a fixed-effects model was applied. Publication bias was detected with an asymmetrical funnel plot and cross-checked by Begg’s and Egger’s tests. The trim-and-fill method was used if publication bias existed. Subgroup analysis was conducted based on the definition of radiographic KSD relapse to reduce the impact of heterogeneity. All data were analyzed by STATA software version 12.0 (Stata Corporation, College Station, TX, USA). *P* < 0.05 was considered statistically significant.

## Results

### Study characteristics

**Fig. 1:**
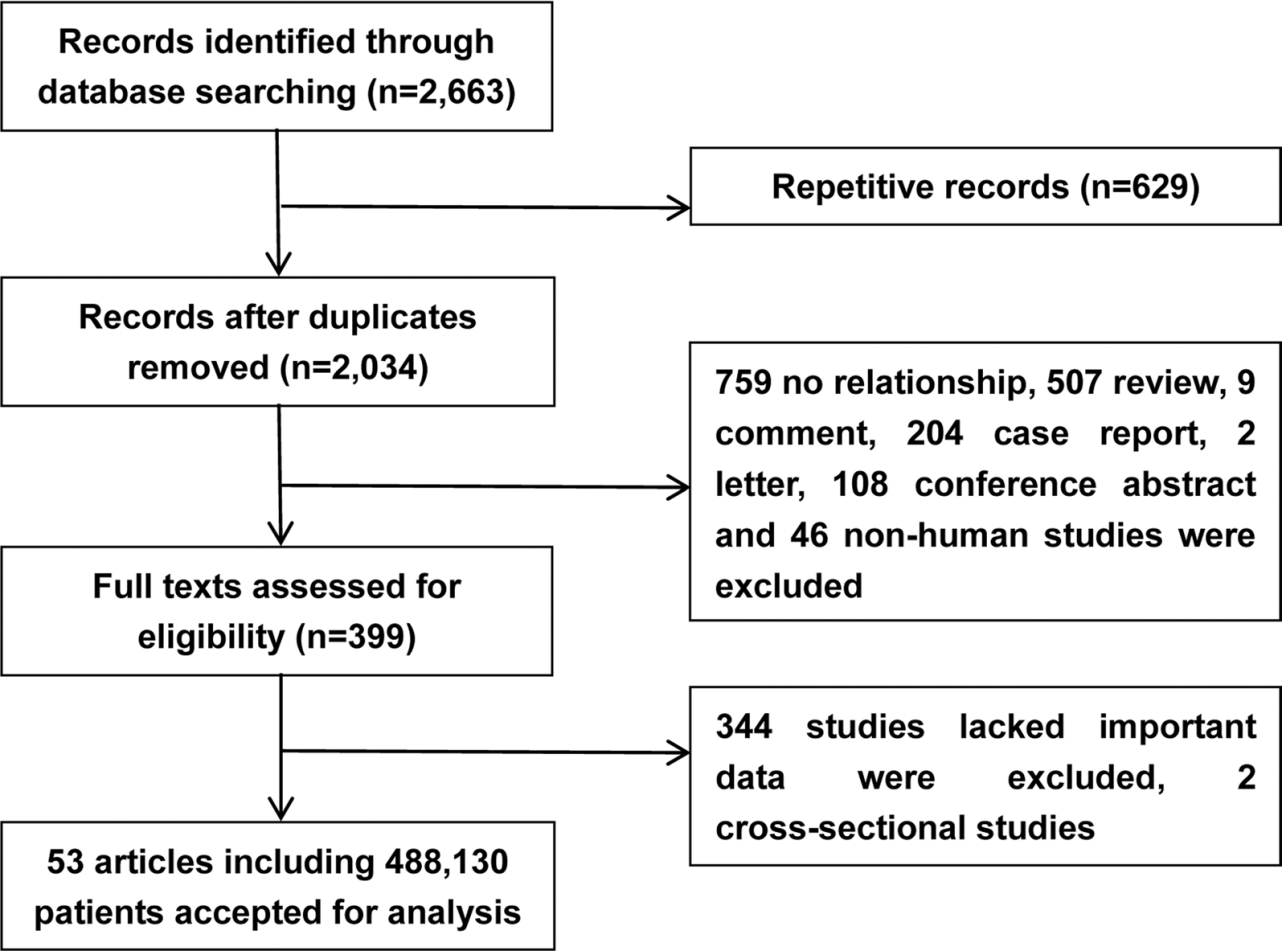
Flow diagram of the study selection process

First, a total of 2,663 records (PubMed: 1,561; Embase: 207; Web of Science: 940) were collected. A total of 399 articles were further evaluated carefully after deduplication and reviewing the title and abstracts. A total of 344 studies were further excluded, which lacked important data. 2 cross-sectional studies were also excluded. Eventually, 53 articles, including 488,130 patients, were enrolled in this analysis [6, 16–67] (Fig. 1). These patients were from the USA (94.90%), Japan (2.80%), China (0.57%), Italy (0.55%), Korea (0.52%), Egypt (0.16%), Germany (0.13%), Israel (0.09%), Turkey (0.08%), Spain (0.05%), Canada (0.04%), France (0.04%), Iceland (0.04%), Belgium (0.02%), and Sweden (0.01%).

The characteristics of these enrolled studies are shown in Table 1. Approximately 17.4% of patients enrolled in this study experienced the recurrence of KSD. The patients in four studies [26, 35, 42, 43] were from the same research institutions. However, the collection time and the risk factors they reported were not exactly the same. Thus, these four studies were still included in this meta-analysis. Additionally, two other researches [18, 28] may have the same cohort. After comparison, we screened the possible duplicate data and retained which item had more participants. There were 40 retrospective studies and 13 prospective studies enrolled in our analysis. Populations from Caucasian, Asian and mixed races were reported in 20, 14, and 19 studies, respectively.

**Table 1:** Main characteristics of all studies included in this analysis

Study	Nation	Research type	Time data collected	Sampling frame	Follow-up time	Stone types	Race	Age	Sample size	Man (%)	Recurrence rate (%)	Ratio
Song et al. [16]	USA	Retrospective	2007–2013	SC	Median 64 m	NA	Mixed	Mean 57.6	14,854	93.81	57.60	HR
Ito et al. [17]	Japan	Retrospective	2012–2019	SC	Median 31 m	Mixed	Asian	Mean 60.0	664	63.00	20.33	HR
Iremashvili et al. [18]	USA	Retrospective	2009–2017	SC	Mean 4.3 y	NA	Mixed	Mean 54.9	1,970	51.62	20.96	HR
Samson et al. [19]	USA	Retrospective	2007–2017	SC	3 y	NA	Mixed	Mean 46.0	434,055	57.69	14.50	OR
Prasanchaimontri and Monga [20]	USA	Retrospective	2002–2012	SC	Median 10 y	Mixed	Mixed	NA	1,617	62.71	23.07	OR
Nevo et al. [21]	Israel	Retrospective	2010–2015	SC	Median 38 m	Mixed	Caucasian	Median 53	457	69.20	24.29	HR
Islam et al. [22]	USA	Retrospective	2008–2018	SC	10 y	NA	Mixed	Mean 57.6	69	44.93	23.19	OR
Ingvarsdottir et al. [23]	Iceland	Retrospective	1985–2013	SC	Median 12 y	Mixed	Caucasian	Median 15	190	41.05	35.79	HR
Castiglione et al. [24]	USA	Prospective	2009	SC	5 y	NA	Mixed	Mean 48.2	375	54.80	21.07	OR
Vaughan et al. [25]	USA	Retrospective	1984–2012	SC	NA	NA	Mixed	Mean 43.9	3,364	60.79	26.22	HR
Kang et al. [26]	Korea	Retrospective	1994–2017	SC	15 years	NA	Asian	Mean 49.1	680	60.15	41.18	HR
Iremashvili et al. [27]	USA	Retrospective	2009–2016	SC	Median 4.8 y	NA	Mixed	Mean 53.6	498	52.21	17.67	HR
Iremashvili et al. [28]	USA	Retrospective	2009–2017	SC	Mean 4.1 y	NA	Mixed	Mean 54.8	1,496	52.07	24.53	HR
Ruysscher et al. [29]	Belgium	Retrospective	1998–2016	SC	NA	NA	Caucasian	Median 3.9	97	73.20	34.02	OR
Costa et al. [30]	USA	Prospective	2009–2013	SC	5 y	Mixed	Mixed	Mean 49.6	175	53.14	66.86	OR
Yamashita et al. [31]	Japan	Retrospective	2011–2015	SC	NA	NA	Asian	Median 59	300	69.33	49.33	OR
Wang et al. [32]	China	Retrospective	2015	SC	NA	COS	Asian	mean 50.6	72	75.00	50.00	OR
Ozgor et al. [33]	Turkey	Retrospective	2011–2013	SC	Mean 33.3 m	Mixed	Caucasian	Mean 47.33	202	53.11	22.28	OR
Ferraro et al. [34]	Italy	Prospective	1993–1994	SC	5 y	COS	Caucasian	Mean 45.3	103	NA	33.98	HR
Tasian et al. [6]	USA	Retrospective	2008–2014	SC	3 y	Mixed	Caucasian	Median 14.8	285	45.61	23.86	HR
Kang et al. [35]	Korea	Retrospective	1994–2015	SC	NA	Mixed	Asian	Mean 44.9	624	63.58	37.66	HR
Shih et al. [36]	China	Retrospective	2000–2002	SC	Mean 8.9 y	NA	Asian	Mean 27.48	1,474	0.00	16.62	HR
Guerra et al. [37]	Italian	Retrospective	1986–2013	SC	NA	CS	Caucasian	NA	2,080	61.01	6.11	OR
El-Assmy et al. [38]	Egypt	Retrospective	1998–2011	SC	10 y	Mixed	Caucasian	Mean 41.3	784	73.09	25.26	HR
Bos et al. [39]	Canada	Prospective	2009–2010	SC	5 y	NA	Caucasian	Mean 54.5	110	63.64	25.45	HR
Liu et al. [40]	China	Retrospective	1999–2010	SC	NA	NA	Asian	Mean 52.8	1,259	85.94	13.26	HR
Rule et al. [41]	USA	Retrospective	1984–2003	SC	NA	Mixed	Mixed	Mean 41.7	2,239	62.48	31.58	HR
Kang et al. [42]	Korea	Retrospective	1994–2010	SC	Median 35 m	Mixed	Asian	NA	240	NA	23.33	HR
Kang et al. [43]	Korea	Retrospective	2007–2011	SC	NA	Mixed	Asian	Mean 60.4	342	48.25	16.96	HR
Kruck et al. [44]	Germany	Retrospective	2001–2007	SC	NA	Mixed	Caucasian	Mean 51.5	482	66.00	NA	OR
Kohjimoto et al. [45]	Japan	Retrospective	2005	MC	7 y	Mixed	Asian	Mean 52.5	11,555	73.86	57.14	OR
Sorensen et al. [46]	USA	Retrospective	2001–2010	SC	NA	NA	Caucasian	Mean 55	40	32.50	22.50	OR
Pieras et al. [47]	Spain	Retrospective	2003–2007	SC	Mean 60 m	Mixed	Caucasian	Mean 44	248	69.76	48.79	HR
Ha et al. [48]	Korea	Retrospective	1994–2008	SC	NA	CS	Asian	NA	247	NA	39.68	HR
DeFoor et al. [49]	USA	Retrospective	1999–2006	SC	NA	Mixed	Mixed	Mean 12.7	139	52.52	36.69	OR
Kim et al. [50]	Korea	Retrospective	1994–2007	SC	Median 49 m	CS	Asian	mean 44.3	266	65.20	41.73	HR
Lee et al. [51]	Korea	Retrospective	1996–2006	SC	Median 54 m	Mixed	Asian	Mean 42.9	163	66.76	36.20	HR
Krambeck et al. [52]	USA	Retrospective	1983–1984	SC	> 5 y	Mixed	Mixed	NA	375	64.80	49.60	OR
Unal et al. [53]	Turkey	Retrospective	NA	SC	NA	NA	Caucasian	Mean 35	173	50.87	28.32	HR
Daudon et al. [54]	France	Retrospective	1984–2000	SC	3 y	COS	Caucasian	Mean 30.4	181	70.17	39.78	HR
Abe et al. [55]	Japan	Retrospective	1987–2000	SC	5 y	Mixed	Asian	Mean 45.7	11,39	72.10	28.62	OR
Parks et al. [56]	USA	Prospective	1970–2003	SC	30 y	ICN	Mixed	Mean 33.0	1,201	70.86	NA	HR
Mardis et al. [57]	USA	Prospective	1995–1996	SC	7 y	Mixed	Mixed	NA	203	70.44	29.06	HR
Afshar et al. [58]	Canada	Retrospective	1990–2002	SC	Mean 46 m	Mixed	Caucasian	Mean 7	83	46.99	31.33	OR
Siener et al. [59]	Germany	Prospective	NA	SC	2 y	COS	Caucasian	Mean 51.7	134	67.16	42.54	OR
Chen et al. [60]	USA	Retrospective	1973–1996	SC	5 y	NA	Mixed	Mean 37	62	87.10	30.65	RR
Borghi et al. [61]	Italy	Prospective	1993–1994	SC	5 y	COS	Caucasian	Mean 45.1	120	100.00	43.33	RR
Jendle-Bengten et al. [62]	Sweden	Retrospective	NA	SC	Mean 5.6 y	COS	Caucasian	Mean 50	52	73.08	51.92	HR
Trinchieri et al. [63]	Italy	Prospective	1980–1990	SC	Mean 19.3 y	Mixed	Caucasian	Mean 44.3	195	50.26	26.67	HR
Ettinger et al. [64]	USA	Prospective	NA	SC	3 y	COS	Mixed	Mean 48.0	64	78.13	39.06	RR
Hiatt et al. [65]	USA	Prospective	1984–1985	MC	4.5 y	COS	Mixed	Mean 43.0	99	78.79	14.14	HR
Gambaro et al. [66]	Italy	Prospective	1984–1986	SC	9 y	NA	Caucasian	Median 34	190	65.79	57.89	OR
Streem [67]	USA	Prospective	1983	SC	Mean 41.7 m	MAPS	Mixed	Mean 53.2	44	20.45	27.27	OR

SC, single center; MC, multi-center; NA, not available; OR, odds risk; RR, relative risk; HR, hazard risk; CS, calcium stone; COS, calcium oxalate stone; MAPS, magnesium-ammonium calcium phosphate stone; y, year; m, month

### Quality assessment

All the studies included in this meta-analysis were assessed according to the NOS. The average quality score of the studies was 7.8 (ranging from 5 to 9). All the studies including 48 high-quality and 5 moderate-quality studies were performed using an improved methodology. For further analysis, all the studies mentioned above were enrolled.

### Demographic risk factors

Eleven variables, including age [6, 16–18, 20–22, 25–27, 31, 32, 36, 41, 42, 47, 48, 50], body mass index (BMI) [6, 17, 18, 20–23, 25, 26, 29, 32, 35, 42, 45, 46, 51], sex [6, 16, 17, 20, 21, 23, 25–28, 31, 32, 35, 40–42, 45, 46, 48, 50, 59, 63, 66], race [18, 27, 41], pregnant or childbirth [25, 36], gout [16, 18, 40], diabetes [16, 18, 31, 40, 45], hypertension [16, 18, 31, 40, 45], hyperlipidemia [31, 40, 45], osteoporosis [16, 40], and urinary tract anomalies [59, 67] were available for data pooling (Table 2).

**Table 2:** The pooled relationship between various risk factors and relapse of kidney stone disease

Risk factors	No. of studies	No. of patients	OR (95% CI)	*P* value	Model	Heterogeneity	
						*I*^2^(%)	*P*
**Demographic risk factors**							
Age	18	28,315	0.980 (0.966–0.995)	0.009^#^	Random	84.7	< 0.001^§^
BMI	16	22,087	1.045 (1.008–1.083)	0.016*	Random	62.4	< 0.001^§^
Sex	23	41,466	1.046 (0.945–1.157)	0.388	Random	65.8	< 0.001^§^
Race	3	4,707	1.338 (1.033–1.732)	0.027*	Fixed	0.0	0.982
Pregnant or childbirth	2	3,609	0.896 (0.228–3.525)	0.875	Random	96.8	< 0.001^§^
Gout	3	18,083	1.181 (0.745–1.871)	0.479	Random	79.4	0.008^#^
Diabetes	5	29,938	1.095 (0.959–1.251)	0.179	Random	56.3	0.058
Hypertension	5	29,938	1.126 (1.076–1.178)	< 0.001^§^	Fixed	0.0	0.579
Hyperlipidemia	3	13,114	1.020 (0.670–1.553)	0.925	Random	74.4	0.020*
Osteoporosis	2	16,113	1.140 (0.743–1.749)	0.550	Random	52.5	0.147
Urinary tract anomalies	2	178	1.098 (0.274–4.405)	0.895	Random	65.8	0.087
**Kidney stone-related risk factors**							
Family history of kidney stones	12	11,912	1.194 (1.078–1.323)	0.001^#^	Random	46.8	0.037*
Personal history of kidney stones	11	10,784	1.428 (1.230–1.658)	< 0.001^§^	Random	52.1	0.022*
Any gross hematuria with first symptomatic stone	2	2,737	1.068 (0.893–1.276)	0.473	Fixed	0.0	0.324
Suspected kidney stone episodea prior to first confirmed stone episode	3	6,101	1.815 (1.559–2.114)	< 0.001^§^	Fixed	0.0	0.802
Any concurrent asymptomatic (nonobstructing) stone	2	2,737	1.711 (1.464–1.999)	< 0.001^§^	Fixed	2.0	0.312
Uric acid stone	4	4,602	1.957 (1.414–2.707)	< 0.001^§^	Fixed	40.0	0.172
Calcium oxalate monohydrate	2	3,612	0.897 (0.785–1.025)	0.110	Fixed	0.0	0.331
Calcium phosphate stone	2	1,865	1.271 (0.592–2.731)	0.538	Fixed	37.2	0.207
Diameter of largest kidney stone	8	3,771	1.047 (0.995–1.101)	0.076	Random	74.4	< 0.001^§^
Multiple calculi	4	1,760	1.338 (0.965–1.855)	0.080	Random	80.3	0.002^#^
Bilateral nephrolithiasis	2	2,218	2.175 (0.860–5.500)	0.101	Random	82.2	0.018*
Pelvic or lower pole kidney stone	3	6,101	1.666 (1.264–2.195)	< 0.001^§^	Random	76.6	0.014*
Ureteral stone	2	1,387	0.888 (0.380–2.075)	0.785	Random	85.7	0.008^#^
Ureterovesical junction stone	3	6,101	0.845 (0.761–0.937)	0.001^#^	Fixed	0.0	0.439
**Treatment method risk factors**							
Stone prevention medications	9	4,316	0.752 (0.548–1.033)	0.078	Random	76.0	< 0.001^§^
Potassium citrate	4	2,992	0.732 (0.345–1.554)	0.417	Random	87.7	< 0.001^§^
Surgery	3	8,23	2.161 (1.557–2.998)	< 0.001^§^	Fixed	0.0	0.457
ESWl	4	1,495	1.756 (0.606–5.086)	0.299	Random	93.9	< 0.001^§^
**24-h urine and serum tests related risk factors**							
Baseline urine volume	6	1,789	0.934 (0.756–1.154)	0.528	Random	64.0	0.016*
Baseline urine calcium	8	2,552	1.001 (0.997–1.005)	0.531	Random	55.9	0.026*
Baseline low urine citrate	7	2,371	1.000 (0.998–1.002)	0.994	Random	55.6	0.035*
Baseline urine oxalate	7	2,371	0.999 (0.993–1.004)	0.675	Fixed	26.3	0.228
Baseline urine sodium	4	1,719	1.001 (0.999–1.002)	0.325	Fixed	0.0	0.563
Baseline urine uric acid	6	2,232	1.000 (0.999–1.001)	0.992	Random	51.1	0.069
Baseline urine magnesium	3	1,095	1.081 (0.777–1.503)	0.645	Fixed	0.0	0.780
Baseline urine phosphate	2	422	0.978 (0.315–3.038)	0.969	Random	89.4	0.002^#^
Baselin urine osmolality	2	855	1.257 (0.629–2.515)	0.517	Random	83.3	0.014*
CaOx SS (DG)	2	314	0.808 (0.611–1.068)	0.134	Fixed	0.0	0.972
Serum calcium	2	348	1.033 (0.787–1.356)	0.817	Fixed	0.0	0.790
GFR	3	1,094	1.017 (0.963–1.074)	0.539	Random	92.3	< 0.001^§^
24 h urine test completion	2	448,909	1.157 (1.128–1.186)	< 0.001^§^	Fixed	0.0	0.519

BMI, body mass index; OR, odds ratio; CI, confidence intervals; ESWl, extracorporeal shock wave lithotripsy; SS, supersaturation; DG, delta Gibb’s free energy; GFR, glomerular filtration rate

*P < 0.05; ^#^*P* < 0.01; ^§^*P* < 0.001

The pooling data suggested that the patients with older age would have a lower risk for recurrence of KSD. Caucasian and the patients with higher BMI or hypertension would have a higher risk for recurrence of KSD (Additional file 2: Figure S1). Meanwhile, sex, pregnant or childbirth, gout, diabetes, hyperlipidemia, osteoporosis, or urinary tract anomalies might not be the risk factors for recurrence of KSD. No publication bias appeared.

### Kidney stone-related risk factors

Fourteen variables including family history of nephrolithiasis [18, 22, 25, 27, 35, 37, 41, 42, 48, 50, 54, 59], personal history of nephrolithiasis [18, 25, 27, 29, 38, 39, 41, 48, 51, 53, 55], any gross hematuria with first symptomatic stone [27, 41], suspected nephrolithiasis episode a prior to first confirmed stone episode [25, 27, 41], any concurrent asymptomatic (nonobstructing) stone [27, 41], uric acid stone [20, 27, 41, 47], calcium oxalate monohydrate stone [25, 47], calcium phosphate stone [20, 47], diameter of largest nephrolithiasis [17, 21, 32, 38, 44, 53, 55], multiple stones [42, 48, 55, 59], bilateral nephrolithiasis [18, 47], pelvic or lower pole nephrolithiasis [25, 27, 41], ureteral stone [47, 55], and ureterovesical junction stone [25, 27, 41] were available for data pooling (Table 2). Personal history of nephrolithiasis was defined as the nephrolithiasis history prior to the medical records investigated.

The pooling data suggested that the patients with family history of nephrolithiasis, personal history of nephrolithiasis, suspected nephrolithiasis episode a prior to first confirmed stone episode, any concurrent asymptomatic (nonobstructing) stone, pelvic or lower pole nephrolithiasis, or uric acid stone would have a higher risk for recurrence of KSD (Additional file 2: Figure S2). Additionally, patients with ureterovesical junction stone might have a lower risk in KSD recurrence. Meanwhile, any gross hematuria with first symptomatic stone, calcium oxalate monohydrate stone, calcium phosphate stone, diameter of largest nephrolithiasis, multiple stones, bilateral nephrolithiasis or ureteral stone might not be the risk factors for recurrence of KSD.

The *P* value of Egger’s test of the diameter of largest nephrolithiasis was 0.01. After being adjusted with the method of trim-and-fill, the pooled data was still not statistically significant (OR = 1.024, 95% CI = 0.963–1.089, *P* = 0.456). Thus, the pooled result for diameter of largest nephrolithiasis was reliable. No publication bias appeared in other analysis of risk factors.

### Treatment method related risk factors

Three variables containing stone prevention medications treatment, surgery treatment and extracorporeal shock wave lithotripsy (ESWL) were available for data pooling (Table 2).

#### Stone prevention medications

The pooling data from 7 articles [17, 20, 21, 40, 57, 62, 64] including 9 studies containing 4,316 patients suggested that being treated with stone prevention medications may not lower the risk of KSD recurrence (*I*^2^ = 76.0%, *P* < 0.001; OR = 0.752, 95% CI = 0.548–1.033, *P* = 0.078) (Table 2). No publication bias appeared.

Additionally, we pooled the data from 4 studies [20, 40, 62, 64] reporting the risk factor of potassium citrate. The results showed that treatment with potassium citrate may not lower the risk of KSD recurrence (*I*^2^ = 87.7%, *P* < 0.001; OR = 0.732, 95% CI = 0.345–1.554, *P* = 0.417) (Table 2). The publication bias did not exist.

#### Surgery versus conservative treatment

The pooling data from 3 studies [17, 29, 60] containing 823 patients suggested that the patients need to be treated with surgery would have a higher risk for recurrence of KSD (*I*^2^ = 0.0%, *P* = 0.457; OR = 2.161, 95% CI = 1.557–2.998, *P* < 0.001) (Additional file 2: Figure S3A). No publication bias appeared.

#### ESWL versus other treatment

The pooling data from 4 studies [33, 38, 52, 59] containing 1,495 patients suggested that being treated with ESWL may not lower the risk of KSD recurrence (*I*^2^ = 93.9%, *P* < 0.001; OR = 1.756, 95% CI = 0.606–5.086, *P* = 0.299) (Table 2). The *P* value of Egger’s test was 0.015. After being adjusted with the trim-and-fill method, the pooled data was still not statistically significant (OR = 0.696, 95% CI = 0.265–1.828, *P* = 0.462). Thus, the pooled result for ESWL was reliable.

### 24-h urine and serum tests related risk factors

Eleven variables of 24-h urine test including baseline urine volume [26, 30, 42, 48, 50, 54], baseline urine calcium [26, 30, 35, 42, 48–50, 54], baseline low urine citrate [26, 30, 35, 42, 48–50], baseline urine oxalate [26, 30, 35, 42, 48–50], baseline urine sodium [26, 30, 35, 42], baseline urine uric acid [26, 30, 35, 42, 48, 50], baseline urine magnesium [26, 30, 42], baseline urine phosphate [30, 48], baseline urine osmolality [26, 30], CaOx Supersaturation (SS) delta Gibb’s free energy (DG) [30, 49], and 24 h urine test completion [16, 19] were available for data pooling. Besides, two variables, serum tests containing serum calcium [30, 53] and glomerular filtration rate (GFR) [26, 32, 42], were also obtained. Baseline urine was defined as the urine collected when the patient saw a doctor at the first time [54].

After pooling the data of the risk factors mention above, 24 h urine test completion was suggested to be a risk factor for recurrence of KSD (Additional file 2: Figure S3B). Besides, none of them might be risk factors for KSD recurrence (Table 2). No publication bias appeared.

### Other risk factors

**Fig. 2:**
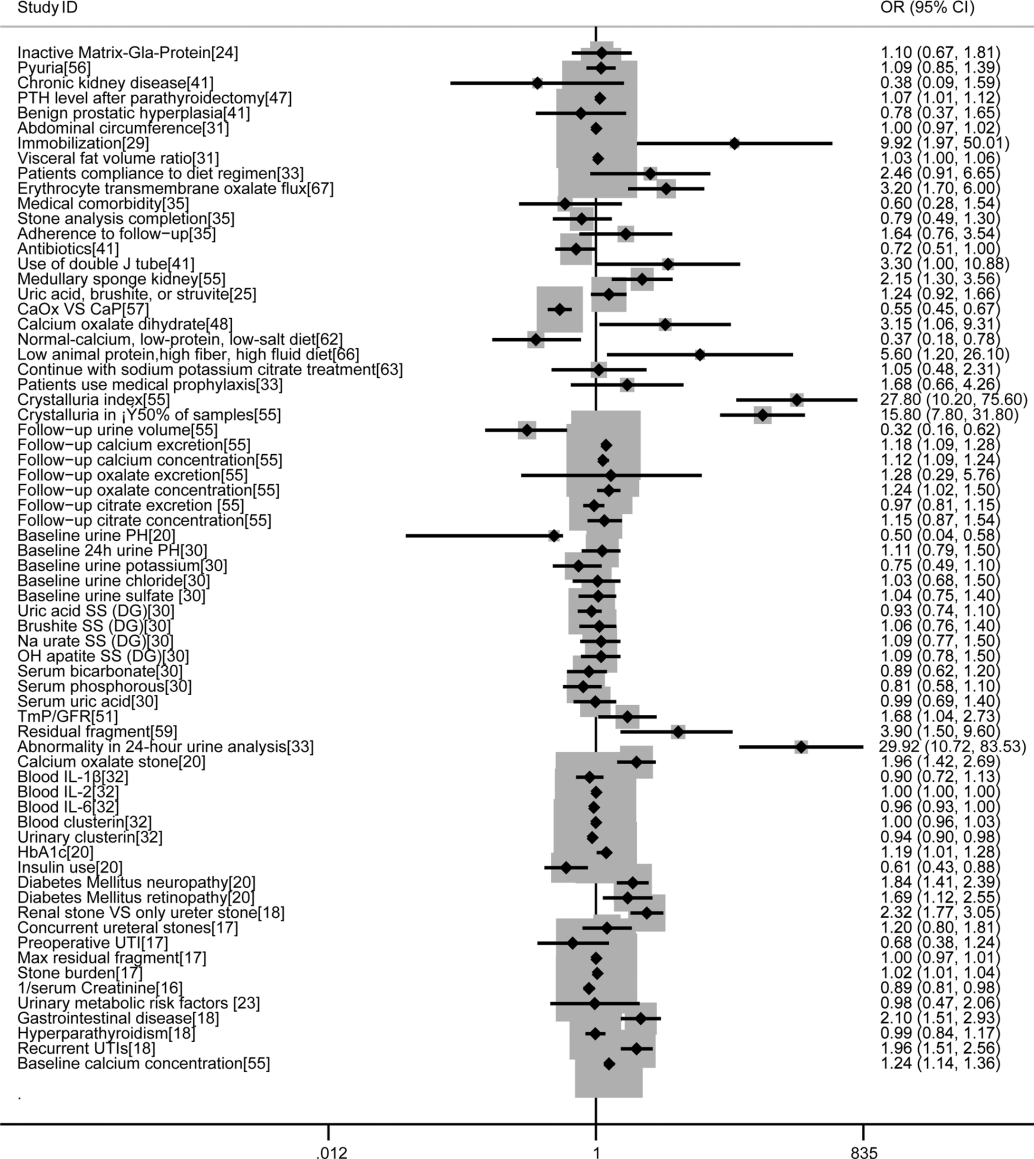
Forest plots of risk factors only reported in one study for KSD relapse respectively

There were 68 risk factors for recurrence of KSD only reported in only one study. As a reference for future research, we listed them in Fig. 2 to make them more intuitive. Follow-up urine was defined as the urine collected during the follow-up [54].

### Subgroup analysis

To reduce the impact of heterogeneity between the studies identified, 30 studies [20, 21, 26, 29, 30, 33–35, 38, 42, 43, 45, 46, 48, 50, 51, 53–56, 58–67] which reported the definition of radiographic KSD relapse were further analyzed (Table 3). The risk factors of higher BMI, personal history of nephrolithiasis, and surgery were still significant.

**Table 3:** The pooled relationship between various risk factors and any radiographic relapse of kidney stone disease

Risk factors	No. of studies	No. of patients	OR (95% CI)	*P* value	Model	Heterogeneity	
						*I*^2^ (%)	*P*
**Demographic risk factors**							
Age	6	5,020	0.996 (0.971–1.022)	0.762	Random	79.5	< 0.001^§^
BMI	9	15,473	1.062 (1.015–1.111)	0.009^#^	Random	63.6	0.005^#^
Sex	12	16,245	1.128 (0.976–1.305)	0.104	Random	56.6	0.008^#^
Urinary tract anomalies	2	178	1.098 (0.274–4.405)	0.895	Random	65.8	0.087
**Kidney stone-related risk factors**							
Family history of kidney stones	6	1,692	1.089 (0.966–1.227)	0.162	Fixed	0.0	0.830
Personal history of kidney stones	5	2,506	1.402 (1.239–1.587)	< 0.001^§^	Fixed	0.0	0.426
Diameter of largest kidney stone	4	2,553	1.014 (0.999–1.029)	0.059	Fixed	38.5	0.181
Multiple calculi	4	1,760	1.338 (0.965–1.855)	0.080	Random	80.3	0.002^#^
**Treatment method risk factors**							
Stone prevention medications	4	2,190	0.674 (0.421–1.079)	0.100	Random	82.6	0.001^#^
Potassium citrate	3	1733	0.529 (0.221–1.255)	0.148	Random	88.4	< 0.001^§^
Surgery	2	159	3.178 (1.597–6.322)	0.001^#^	Fixed	0.0	0.951
ESWl	3	1,120	1.825 (0.386–8.615)	0.448	Random	94.1	< 0.001^§^
**24-h urine and serum tests related risk factors**							
Baseline urine volume	6	1,789	0.934 (0.756–1.154)	0.528	Random	64.0	0.016*
Baseline urine calcium	7	2,413	1.001 (1.000–1.002)	0.224	Fixed	28.4	0.209
Baseline low urine citrate	6	2,232	1.000 (1.000–1.000)	1.000	Fixed	0.0	0.826
Baseline urine oxalate	6	2,232	0.999 (0.993–1.004)	0.690	Fixed	32.7	0.190
Baseline urine sodium	4	1,719	1.001 (0.999–1.002)	0.325	Fixed	0.0	0.563
Baseline urine uric acid	6	2,232	1.000 (0.999–1.001)	0.992	Fixed	51.4	0.069
Baseline urine magnesium	3	1,095	1.081 (0.777–1.503)	0.645	Fixed	0.0	0.780
Baseline urine phosphate	2	422	0.978 (0.315–3.038)	0.969	Random	89.4	0.002^#^
Baselin urine osmolality	2	855	1.257 (0.629–2.515)	0.517	Random	83.3	0.014*
Serum calcium	2	348	1.033 (0.787–1.356)	0.817	Fixed	0.0	0.790
GFR	2	1,022	1.505 (0.656–3.453)	0.335	Random	95.9	< 0.001^§^

BMI, body mass index; OR, odds ratio; CI, confidence intervals; ESWl, extracorporeal shock wave lithotripsy; GFR, glomerular filtration rate

^*^ *P* < 0.05, ^#^ *P* < 0.01, ^§^*P* < 0.001

## Discussion

This study comprehensively and systematically analyzed the association between various risk factors and the recurrence of KSD. We identified 12 risk factors for predicting the recurrence of KSD. Personal history of nephrolithiasis is vital for identifying the incidence of recurrence. Approximately half of the patients with asymptomatic nephrolithiasis will have symptoms when stones pass during the first stone formation [57]. The 5-year recurrence rate of patients with first-time symptomatic stones is approximately 20% [41]. This rate increases with each additional KSD episode [25].

White race seem to be at a higher risk for KSD than African Americans [68]. Interestingly, our results indicated that Caucasians may undergo more recurrences of KSD than other race patients. It is not exactly known why KSD has a greater recurrence rate in Caucasian, probably because of genetic factors [5]. Thus, clinicians need to take racial differences into account when developing strategies for kidney stone prevention for patients. Younger age may also reflect a genetic component that leads to the early presentation of stones and their recurrence [41].

Family history is associated with a high incidence of KSD, which may also be related to genetic factors. A recent meta-analysis identified 20 nephrolithiasis-associated loci, including CYP24A1, DGKD, DGKH, WDR72, GPIC1, and BCR locus which were predicted to affect vitamin D metabolism and calcium-sensing receptor signaling respectively [69]. Patients with a personal history of KSD, whether symptomatic or asymptomatic, also had an increased risk of recurrence. The recurrence rate increases with each additional kidney stone episode [70]. Furthermore, nonobstructing stones are independent predictors for symptomatic recurrence [41]. If these nonobstructing stones are not treated with surgery, they can pass in the future, become obstructive and then lead to recurrence of symptoms [71].

Obesity, diabetes, hypertension and hyperlipidemia are commonly considered the main clinical characteristics of metabolic syndrome [45]. Metabolic syndrome is related to many kinds of chronic diseases. Epidemiological survey points out that the prevalence of metabolic syndrome is increasing which affects almost a quarter of European population [72]. It is also considered to elevate the rate of nephrolithiasis formation [73]. The KSD patients with higher BMI are easier to experience recurrence in our study as well. A meta-analysis containing 13 cohort studies clarified that relative risk of kidney stones for a 5-unit increment in BMI was 1.21 (1.12–1.30) [74]. In addition, hypertension was also identified as a risk factor for KSD recurrence. This is an important finding because the mechanism of hypertension promoting renal stone formation and recurrence remains unclear. Only a few studies have examined the underlying mechanisms between them. Liu et al,. reported that changes in the blood pressure have a direct consequence on the urinary microbiome and this effect could promote the formation of KSD [75]. Therefore, the control and monitoring of blood pressure is necessary for prevention of KSD recurrence. This is also an important finding of this meta-analysis.

Patients requiring surgery also have a higher risk of KSD recurrence. Common surgical procedures for upper urinary calculi are multitudinous. We believe that compared with the patients receiving conservative treatment, the patients accepting surgery have more complex stone situations, including multiple stones or larger diameter of stone [27]. Pelvic or lower pole stones may contribute to the onset of symptoms in the future, as they may be the stones that have previously detached or formed from residual fragments after surgery [76]. Uric acid stone accounts for about 8% of all stone types [77]. Symptomatic recurrence rate for uric acid at 10 years was approximately 50% which is higher than calcium oxalate and hydroxyapatite stones significantly [78]. These data suggested the importance of stone composition analysis in first-time stone formers.

The American Urologic Association Guidelines and European Association of Urology Guidelines stated that 24-h urine was important for high-risk stone formers [9, 79]. Low volume and high urine concentration are both regarded as risk factors for the formation of nephrolithiasis [80]. Thus, higher fluid intake is recommended in current guidelines, but 24-h urine indexes contribution to our analysis were too weak [9, 79]. Nevertheless, patients who completed a 24-h urine test seemed to have a relatively high KSD recurrence rate. One interpretation is that the patients with more significant KSD are more likely to receive metabolic evaluation including 24-h urine [16]. Considering that the 24-h urine is only a test method, the completion of this test itself should not affect the recurrence of stones. Preventive interventions based on 24-h urine test results do not appear to be working. Considering the evidence for empirical treatment in reducing stone recurrence and the lack of evidence for management based on 24-h urine test outcomes to reduce stone recurrence, Samson et al. suggest that clinicians should consider what results are useful [19]. They questioned whether those providers interpreted 24-h urine test results or counseled patients effectively, or whether patients followed the recommendations.

Potassium citrate is generally considered a relatively safe and commonly used prophylactic for preventing stone recurrence [81]. The treatment of potassium citrate in this study did not seem to reduce the recurrence rate. This This may be related to being affected by the result from Liu et al. [40]. In their research, patients prescribed potassium citrate increased risk of recurrence. They thought that this result might be associated with confounding by indication.

To the knowledge of us, this is the largest and the most comprehensive meta-analysis to explore the risk factors on KSD recurrence. We tried our best to systematically collect and evaluate high quality researches which reported the risk factors for KSD recurrence. This is also the first meta-analysis demonstrate that hypertension, race, 24 h urine test completion, and ureterovesical junction stone are related to KSD recurrence. We are also the first to comprehensively explore the risk factor for radiographic KSD relapse.

There were still some limitations in this study. First, the data of risk factors for recurrence of KSD used in this analysis were reported directly in the articles enrolled. Part of the data were extracted from KM curves. Second, the follow-up times recorded in these enrolled articles were different. Third, only the studies reporting OR, HR or RR were enrolled. Finally, publication bias existed in two risk factors, which could influence our results. The study on this topic is currently very restricted. More well-designed studies exploring the risk factors for relapse of KSD are still required in the future.

## Conclusion

12 risk factors including younger age, higher BMI, race of Caucasian, family history of nephrolithiasis, personal history of nephrolithiasis, suspected nephrolithiasis episode prior to first confirmed stone episode, any concurrent asymptomatic (nonobstructing) stone, hypertension, uric acid stone, pelvic or lower pole nephrolithiasis, surgery, and 24 h urine test completion were identified to be associated with relapse of KSD. Additionally, the patients with ureterovesical junction stone might have a lower risk in the relapse of KSD. These results could serve as the risk factors for constructing recurrence prediction models. It also supplied a basis for preventing the recurrence of KSD. Although all conclusions were obtained from results of this analysis directly, several risk factors should be interpreted with caution. More well-designed researches on this topic are needed.

## Supplementary Information

**Additional file 1: Table S1.** Keywords and search hits in PubMed, Web of Science, and Embase databases. **Table S2.** Newcastle-Ottawa Quality Assessment Scale for case–control studies. **Table S3.** Newcastle-Ottawa Quality Assessment Scale for cohort studies.

**Additional file 2: Figure S1.** Forest plots of studies evaluating association between identified three demographic risk factors and KSD relapse. **Figure S2.** Forest plots of studies evaluating association between identified nine kidney stone-related risk factors and KSD relapse. **Figure S3.** Forest plots of studies evaluating association between the risk factors of surgery and 24 h urine test completion and KSD relapse.

## Data Availability

All data is fully provided by contacting the corresponding author.

## Abbreviations

KSD: Kidney stone disease
PRISMA: Preferred Reporting Items for Systematic Reviews and Meta-Analyses
MOOSE: Meta-analysis of observational studies in epidemiology
OR: Odds ratio
RR: Relative risk
HR: Hazard ratio
CI: Confidence interval
BMI: Body mass index
NOS: Newcastle–Ottawa Quality Assessment Scale
ESWL: Extracorporeal shock wave lithotripsy
SS: Supersaturation
DG: Delta Gibb’s free energy
GFR: Glomerular filtration rate
SC: Single center
MC: Multi-center
NA: Not available
CS: Calcium stone
COS: Calcium oxalate stone
MAPS: Magnesium-ammonium calcium phosphate stone
y: Year
m: Month
