# Supplementary material for: Risk factors for kidney stone disease recurrence: a comprehensive meta-analysis
Source: BMC Urol. 2022 Apr 19;22:62. doi: 10.1186/s12894-022-01017-4 (PMC9017041; doi:10.1186/s12894-022-01017-4)
Supplement: Supplementary file 1 — Additional file 1: Table S1. Keywords and search hits in PubMed, Web of Science, and Embase databases. Table S2. Newcastle-Ottawa Quality Assessment Scale for case–control studies. Table S3. Newcastle-Ottawa Quality Assessment Scale for cohort studies. [file 12894_2022_1017_MOESM1_ESM.pdf]

Table S1. Keywords and search hits in PubMed, Web of Science, and Embase databases.

Database	Search Terms	Number of Articles
PubMed	((((((((Nephrolith) OR Kidney Calculus) OR Kidney Stones) OR Kidney Stone) OR Renal Calculi)) AND (((((((Relapse) OR Relapses) OR Recurrences) OR Recrudescence) OR Recrudescences)) AND (((((((risk factor) OR association) OR relative risk) OR odds ratio) OR Populations at Risk)	1,561
Web of Science	TS=(Nephrolith OR Kidney Calculus OR Kidney Stones OR Kidney Stone OR Renal Calculi) AND TS=(Relapse OR Relapses OR Recurrences OR Recrudescence OR Recrudescences) AND TS=(risk factor OR association OR relative risk OR odds ratio OR Populations at Risk)	940
Embase	(‘Nephrolith’ OR ‘Kidney Calculus’ OR ‘Kidney Stones’ OR ‘Kidney Stone’ OR ‘Renal Calculi’ (all fields)) AND (‘Relapse’ OR ‘Relapses’ OR ‘Recurrences’ OR ‘Recrudescence’ OR ‘Recrudescences’ (all fields)) AND (‘risk factor’ OR ‘association’ OR ‘relative risk’ OR ‘odds ratio’ OR ‘Populations at Risk’ (all fields))	207

Table S2. Newcastle-Ottawa Quality Assessment Scale for case-control studies.

Study	Selection				Comparability	Outcome			Total
	Is the case definition adequate?(1 point)	Representa-tiveness of the cases(1 point)	Selection of Controls(1 point)	Definition of Controls(1 point)	Comparability of cases and controls on the basis of the design or analysis(2 point)	Ascertain-ment of exposure(1 point)	Same method of ascertainment for cases and controls(1 point)	Non-Response rate(1 point)	
Song 2022[16]	1	1	1	1	2	1	1	1	9
Ito 2021[17]	1	1	1	1	2	1	1	1	9
Iremashvili 2021[18]	1	1	1	1	1	1	1	1	8
Samson 2020[19]	1	1	1	1	2	1	1	1	9
Prasanchaimontri 2020[20]	1	0	1	1	2	1	1	0	7
Nevo 2020[21]	1	1	1	1	2	1	1	1	9
Islam 2020[22]	1	0	1	1	1	1	1	1	7
Ingvarsdottir 2020[23]	1	1	1	1	0	1	1	0	6
Vaughan 2019[25]	1	1	1	1	2	1	0	1	8
Kang 2019[26]	1	1	1	1	2	1	1	1	9
Iremashvili 2019[27]	1	1	1	1	2	1	1	1	9
Iremashvili 2019[28]	1	1	1	0	2	1	1	1	8
Ruysscher 2019[29]	1	1	1	1	2	1	0	1	8
Yamashita 2018[31]	1	1	1	1	2	1	0	1	8
Wang 2018[32]	1	1	1	1	2	1	1	0	8
Ozgor 2018[33]	1	1	1	1	2	1	1	1	9
Tasian 2017[35]	1	1	1	1	2	1	1	1	9
Kang 2017[36]	1	1	1	1	2	1	0	1	8

Shih 2016[37]	1	1	1	1	2	1	1	1	9
Guerra 2016[38]	1	1	1	1	2	1	0	1	8
El-Assmy 2016[39]	1	1	1	1	1	1	1	1	8
Liu 2015[41]	1	1	1	1	2	1	0	1	8
Rule 2014[42]	1	1	1	1	2	1	0	1	8
Kang 2014[43]	1	1	1	1	2	1	1	1	9
Kang 2014[44]	1	1	1	1	1	1	0	1	7
Kruck 2013[45]	1	1	1	1	2	1	0	1	8
Kohjimoto 2013[46]	1	1	1	1	2	1	1	1	9
Sorensen 2012[47]	1	0	1	1	2	1	0	1	7
Pieras 2012[48]	1	1	1	1	2	1	1	1	9
Ha 2010[49]	1	1	1	0	2	1	0	1	7
DeFoor 2010[50]	1	1	1	1	2	1	0	1	8
Kim 2009[51]	1	1	1	1	2	1	1	0	8
Lee 2008[52]	1	1	1	0	2	1	1	1	8
Krambeck 2008[53]	1	1	1	1	2	1	1	1	9
Unal 2005[54]	1	1	1	0	0	1	0	1	5
Daudon 2005[55]	1	1	1	1	2	1	1	1	9
Abe 2005[56]	1	1	1	0	2	1	1	1	8
Afshar 2004[59]	1	1	1	1	2	1	1	1	9
Chen 2002[61]	1	0	1	1	2	1	1	1	8
Jendle-Bengten 2000[63]	1	1	1	1	0	1	1	1	7

Table S3. Newcastle-Ottawa Quality Assessment Scale for cohort studies.

Study	Selection				Comparability	Outcome			Total
	Representativeness of the exposed cohort(1 point)	Selection of the non-exposed cohort(1 point)	Ascertain-ment of exposure(1 point)	Demonstration that outcome of interest was not present at start of study(1 point)	Comparability of cohorts on the basis of the design or analysis(2 point)	Assess-ment of outcome (1 point)	Was follow-up long enough for outcomes to occur(1 point)	Adequacy of follow up of cohorts(1 point)	
Castiglione 2020[24]	1	1	1	1	2	1	1	0	8
Costa 2019[30]	1	1	1	1	2	1	1	0	8
Ferraro 2018[34]	1	1	1	1	2	1	1	0	8
Bos 2016[40]	1	1	0	1	1	1	1	1	7
Parks 2004[57]	1	1	0	1	0	1	1	1	6
Mardis 2004[58]	1	1	1	1	0	1	1	1	7
Siener 2003[60]	1	1	0	1	2	1	1	0	7
Borghi 2002[62]	1	1	0	1	2	1	1	1	8
Trinchieri 1999[64]	1	1	0	1	0	1	1	0	5
Ettinger 1997[65]	1	1	0	1	2	1	1	1	8
Hiatt 1996[66]	1	1	0	1	2	1	1	1	8
Gambaro 1996[67]	1	1	0	1	2	1	1	0	7
Streem 1995[68]	1	1	0	1	0	1	1	0	5
