# Supplementary material for: Risk factors for kidney stone disease recurrence: a comprehensive meta-analysis
Source: BMC Urol. 2022 Apr 19;22:62. doi: 10.1186/s12894-022-01017-4 (PMC9017041; doi:10.1186/s12894-022-01017-4)

Figure S1: Forest plots of studies evaluating association between identified three demographic risk factors and KSD relapse.

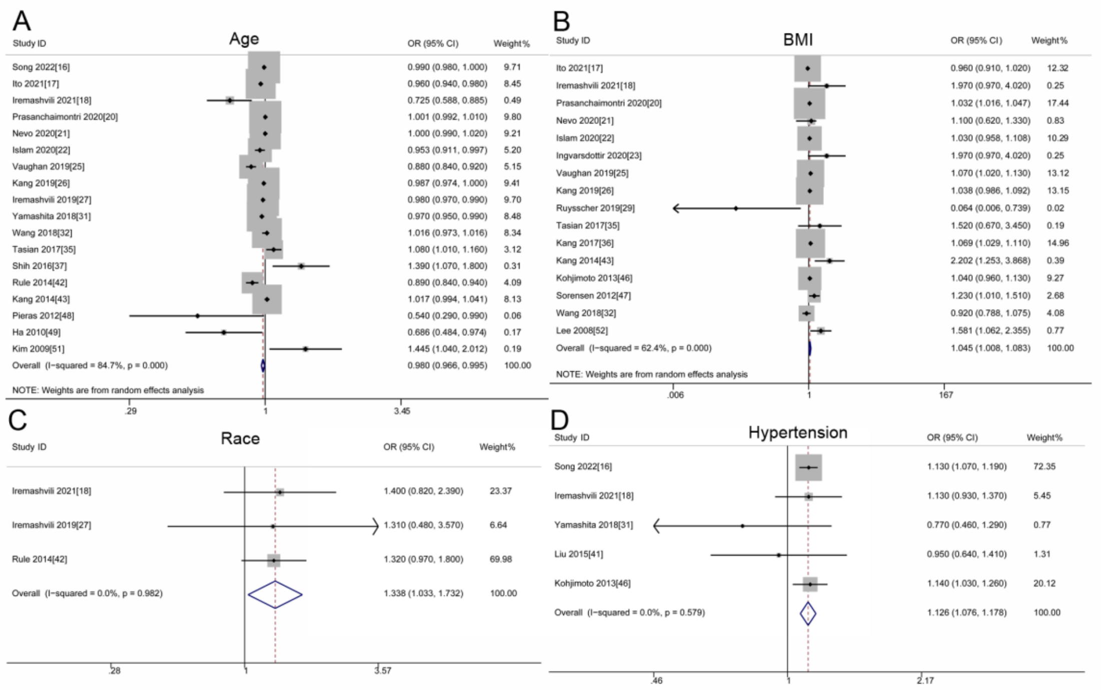

Figure S2: Forest plots of studies evaluating association between identified nine kidney stone-related risk factors and KSD relapse.

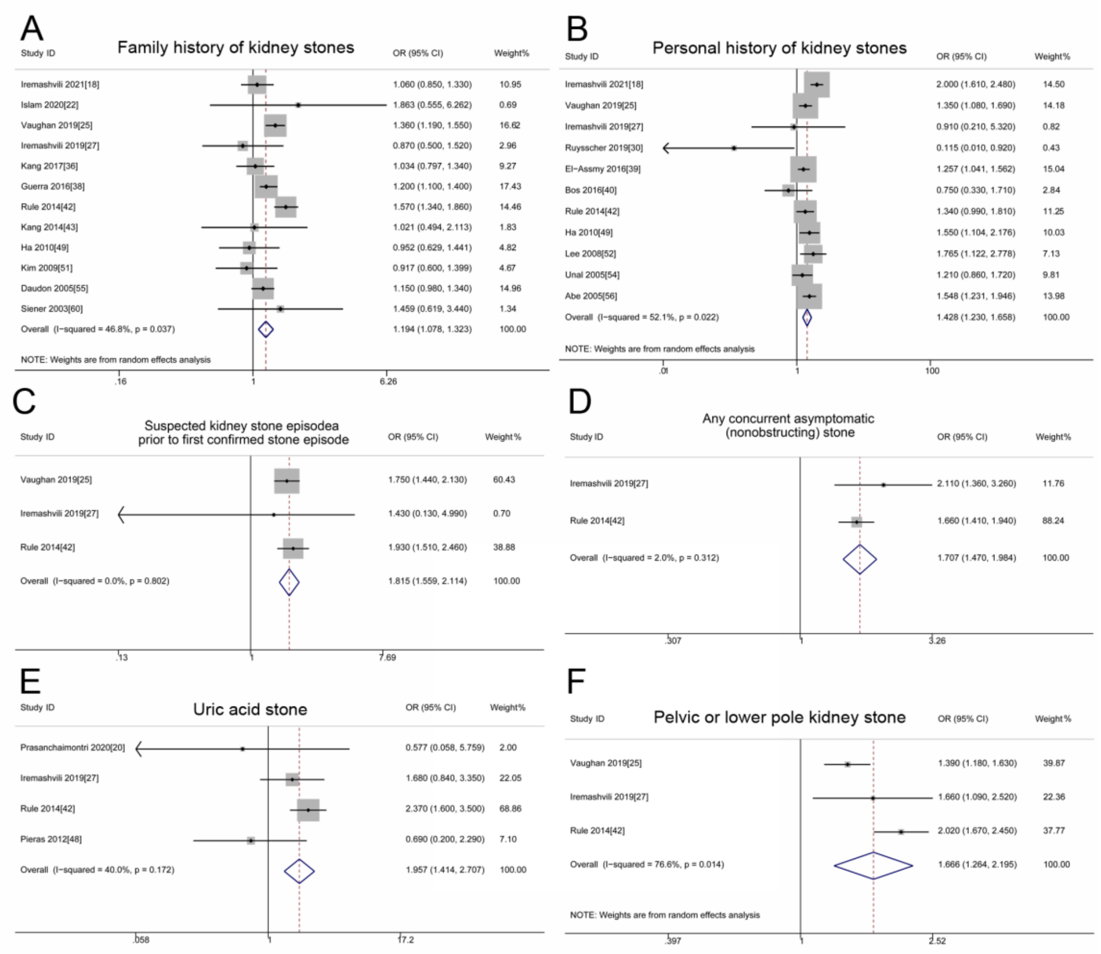

Figure S3: Forest plots of studies evaluating association between the risk factors of surgery and 24h urine test completion and KSD relapse.

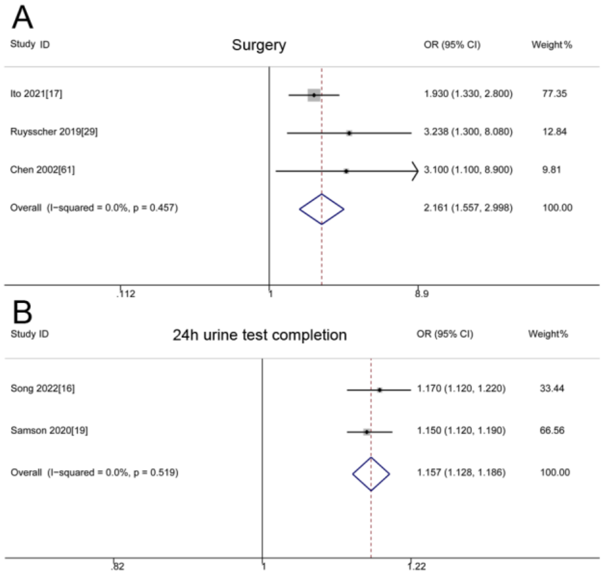

Supplement: Supplementary file 2 — Additional file 2: Figure S1. Forest plots of studies evaluating association between identified three demographic risk factors and KSD relapse. Figure S2. Forest plots of studies evaluating association between identified nine kidney stone-related risk factors and KSD relapse. Figure S3. Forest plots of studies evaluating association between the risk factors of surgery and 24 h urine test completion and KSD relapse. [file 12894_2022_1017_MOESM2_ESM.pdf]
